# Dr. J. Searle Hurlbut

**Published:** 1903-01

**Authors:** 


### DR. J. SEARLE HURLBUT.

DIED of apoplexy, terminating a prolonged attack of Bright's disease, Dr. Jairus Searle Hurlbut, of Springfield, Mass.

Few men in Massachusetts will be more seriously missed from the ranks of dental workers than Dr. Hurlbut. He was not only one of the earnest men in all that pertained to the welfare of his profession, but he was one that possessed broad views of life outside of the comparatively narrow boundaries of dentistry. In whatever field of work he became interested he brought to bear upon it a cultivated intellect. In his personal intercourse with men he exhibited that kindly disposition that resulted in warm and devoted friendships. As a writer said of him, "He had the philosophical temperament, loved nature and his horses, and had learned to throw off the strain of exacting labors when away from the office. Travel had enlarged his outlook. He collected birds; knew where the wild flowers were to be found." Such a man would be out of place in the narrow conventionalities that environ inferior men.

He was active in the organizations of his profession, and became one of the State Board of Registration, being appointed to that position by Governor Ames. From 1891 to 1895 he served as president of the National Association of Dental Examiners. He was a member of the Valley District and the Massachusetts Dental Societies, also of the Northeastern Dental Association and The New York Odontological Society.

Jairus Searle Hurlbut was the son of Asa Hurlbut, who was a descendant of Thomas Hurlbut, a soldier in the Pequot war in 1637. He was born January 5, 1842. He was one of six children. Four out of five brothers studied dentistry, but only two lived to practise any length of time. Dentistry owes much to the family of Hurlbut, for, in addition to these, two of his nephews have made this their life-work.

Dr. Hurlbut graduated from the High School in Springfield in 1860. The next five years were passed in the office with his brother, and in 1865 he went to the Philadelphia Dental College, from which he was graduated. He first located in St. Paul, Minn., where he practised for a year and then returned to Springfield to continue his work. He travelled extensively in this country and Europe, and spent many of his winters in Nassau.

In this world of ours death is inevitable. It is appointed for all men to die, but when the writer is called upon to express the last word for those who have lived truly to the best in their profession, he feels that the silent messenger has all too soon called those to higher activities who are much needed here to uphold the character of the citizen, the profession, and the work of human advancement. Of these, Dr. Hurlbut was a noble exemplar, and over his silent grave the writer would lay the wreath of appreciation and sympathy and recognition of a noble life ended on earth. If this work of Dr. Hurlbut remains as an incentive to his professional brethren to live as he lived, then his death will not have been in vain.

Dr. Hurlbut was maried in 1868 to Miss Julia Ann Sampson. They had no children.

A large concourse of friends, business men, and the dental profession attended Dr. Hurlbut's funeral. He was laid away in Springfield Cemetery.

